# Fortifying Honey: The Effects of Blackcurrant Puree on Functional and Sensory Attributes

**DOI:** 10.1002/fsn3.71769

**Published:** 2026-04-20

**Authors:** Michał Halagarda, Justyna Syguła‐Cholewińska, Marcin Laskoś, Katarzyna Kowa‐Halagarda, Sascha Rohn

**Affiliations:** ^1^ Department of Food Product Quality Krakow University of Economics Krakow Poland; ^2^ Department of Microbiology Krakow University of Economics Krakow Poland; ^3^ Doctoral School Krakow University of Economics Krakow Poland; ^4^ Department of Food Chemistry and Analysis Technische Universität Berlin, Institute of Food Technology and Food Chemistry Berlin Germany

**Keywords:** antioxidant activity, blackcurrant puree, food supplementation, functional foods, honey fortification

## Abstract

Consumers are increasingly looking for foods with enhanced biological activity that positively impact health. Honey is regarded to meet these expectations, because of its traditional use and high biological value, largely attributed to its antioxidant properties and high total phenolic content (TPC). However, honey can also serve as a matrix for the development of different functional formulations and food products to further promote consumers' acceptance and health‐beneficial value, but also for affecting product stability. The present study aimed at evaluating how the incorporation of a phenolic‐rich blackcurrant puree influences the properties of a honey‐based formulation. Physicochemical properties, including antioxidant activity and consumer desirability, were assessed, while microbiological analyses were conducted to evaluate product stability. The results showed that the addition of 7% and 12% blackcurrant puree increased TPC and radical scavenging activity but led to an acidification beyond EU legal limits. In microbiological analyses, no pathogens were detected; however, the possibility of fungal activity could not be excluded. Sensory evaluation indicated that honey with 12% puree was preferred the most, particularly for color and odor. Overall, the study demonstrates that adding frozen fruit as a functional ingredient may be an effective strategy for developing functional honey‐based formulations with enhanced antioxidant potential and improved sensory attractiveness. At the same time, findings highlight key formulation and safety challenges, including acidity management and microbial control, which must be considered when developing fruit‐enriched honey products.

## Graphical abstract

Honey can serve as a matrix for the development of different functional formulations and food products by adding other plant‐based ingredients. This can further promote consumer acceptance and health‐beneficial value, but also improve product stability. In this case, honey was mixed with a phenolic‐rich blackcurrant puree. Physicochemical properties including antioxidant activity and consumer desirability were assessed, while microbiological analyses were conducted to evaluate product stability.

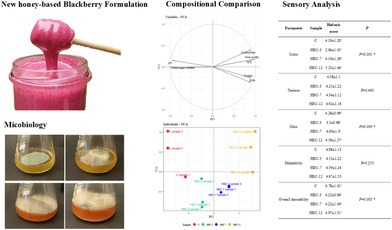

## 1 Introduction

Honey is a natural product with significant nutritional value and traditionally recognized for its potential preventive and therapeutic properties. Despite a reported decline in honey consumption (Kowalczuk et al. 2017), consumer interest in its health‐promoting effects increased in recent decades (Seraglio et al. 2021). The biological activity of honey is hypothesized to be primarily associated with its high antioxidant content, particularly based on phenolic compounds (Zhou et al. 2012; Alvarez‐Suarez et al. 2013; Cianciosi et al. 2018), which are secondary plant metabolites. These compounds are transferred into the honey via the floral nectar (Afrin et al. 2019), propolis, and the pollen (Tomás‐Barberán et al. 2001; Habryka et al. 2020). Due to their chemical structure, phenolic compounds act predominantly as free radical scavengers, with the potential to mitigate the progression of degenerative diseases related to the so‐called oxidative stress (Elamine et al. 2021). Chemically, phenolic compounds are the most effective antioxidants, supporting the recommendations for an increased intake of foods rich in these bioactive substances (Al‐Mamary et al. 2002; Sousa et al. 2016). Notably, Polish honeys have demonstrated substantial diversity in antioxidant activity and phenolic compounds' profiles (Wilczyńska et al. 2005a, 2005b; Wilczyńska 2010; Jasicka‐Misiak et al. 2012; Wesołowska and Dżugan 2017; Jasicka‐Misiak et al. 2017; Dżugan et al. 2018; Pentoś et al. 2020). These variations are largely attributed to the botanical origin of the honey, with buckwheat, heather, and honeydew honeys exhibiting the highest average antioxidant activity and capacity (Wesołowska and Dżugan 2017; Dżugan et al. 2018; Pentoś et al. 2020; Starowicz et al. 2021).

From a regulatory perspective, honey is defined under Council Directive 2001/110/EC as a natural product without added ingredients. Consequently, other foods and formulations containing, for example, fruit components are not allowed to be marketed as honey. At the same time, given the growing consumer demand for the development of products with strong health benefits (Väkeväinen et al. 2021), the question arises whether honey can be combined with other foods possessing high antioxidant properties to further enhance its effects. Although honey already demonstrates considerable antioxidant potential, certain foods, particularly berries, exhibit even stronger properties. Among these, blackcurrants stand out as a popular choice in European markets, being the second most cultivated berry crop (Laaksonen et al. 2014). Notably, Poland leads global exports, accounting for about 80% of frozen blackcurrant exports and 90% of blackcurrant juice concentrate exports (Michalska et al. 2017). This prominence reflects the fruit's nutritional value, as blackcurrants have a high content of anthocyanins and display superior antioxidant activity compared to many other fruits (Alzahrani et al. 2023; Väkeväinen et al. 2021; Gopalan et al. 2012; Jia, Kong, et al. 2012; Jia, Xiong, et al. 2012). In addition to anthocyanins, blackcurrants are a rich source of vitamins A, B group, C, biotin, and folic acid, as well as minerals such as magnesium, calcium, potassium, and iron. They also contain trace elements and organic acids, including iodine, manganese, boron. Additionally, they contain lutein, essential oils, tannins, and pectins. Blackcurrants seem to exhibit even anticancer properties, lower blood cholesterol levels, and inhibit the replication of the influenza virus (Kamanova et al. 2023; Gramza‐Michałowska et al. 2017; Gopalan et al. 2012; Jia, Kong, et al. 2012; Jia, Xiong, et al. 2012). With regard to food products, blackcurrants' phenolic acids are interesting as they demonstrate a range of other beneficial properties, including antibacterial or antiseptic effects (Jessa and Hozyasz 2016). The total phenolic content (TPC) in blackcurrant ranges from 560 to 885.5 mg GAE/100 g (Jessa and Hozyasz 2016).

Given their proven effectiveness as functional ingredients in selected food products, berries also hold promise for improving the health benefits of honey‐based formulations (Andersen et al. 2022; Archaina et al. 2019). Previous attempts have been made to incorporate dried and freeze‐dried fruits into honey. However, studies indicate that these processes lead to the loss of some beneficial properties, particularly a decrease in TPC, with conventional drying causing the most significant degradation (Michalska et al. 2017; Semenov et al. 2015). Furthermore, flavor, color, and texture are modified (Väkeväinen et al. 2021). In contrast, freezing has been shown to better preserve the nutritional value and bioactive compounds of fruits, making it a more effective preservation method in that respect (Semenov et al. 2015; Reyes‐Alvarez and Lanari 2023). Additionally, experiments testing the addition of freeze‐dried (2% and 4%) (Miłek et al. 2023), as well as freeze‐dried and dried (1% and 4%) (Grabek‐Lejko et al. 2022) fruits have been conducted. However, these studies primarily focused on bioactive properties without assessing key physicochemical parameters of honey, its sensory characteristics, or potential microbiological concerns related to fruit ingredient‐derived contaminants. This raises the question of whether the incorporation of frozen fruits at higher concentrations could not only further enhance the antioxidant properties of honey but also affect its overall stability, sensory appearance, and safety aspects, which remain largely unexplored.

In this context, the combination of berries (here: blackcurrants) can also be interesting with regard to microbial stability or synergism of both ingredients. Honey, as a natural product, is exposed to microorganisms of its individual environment. These can enter the honey through the digestive tract of bees, as well as during the collection and transportation of pollen to the hive (Tiusanen et al. 2024; Mattila et al. 2012). The microbiota of honey generally may consist of microorganisms potentially pathogenic to humans, but contamination levels, measured as number of microorganisms per gram product, are typically low. Due to honey's antibacterial properties, associated with several other composition‐related properties (e.g., high sugar concentration, low pH of 3.2–4.5), the production of hydrogen peroxide through the glucose oxidase‐catalyzed oxidation of glucose, presence of antibacterial peptides (such as defensin‐1), many adverse microorganisms are eliminated during the dehydration and maturation processes, while the growth of others may be inhibited (Cianciosi et al. 2018; Kwakman and Zaat 2012). However, spore‐forming bacteria like *Bacillus* spp. and *Clostridium* spp., especially *Clostridium botulinum* , can survive in honey, while the presence of molds such as *Rhizopus* sp., *Curvularia* sp., *Fusarium* sp., *Aspergillus* sp., and others may indicate inadequate hygiene handling during collection and processing or environmental contamination (Rosiak et al. 2020; Grabowski and Klein 2017; Fernandez et al. 2017; Snowdon and Cliver 1996).

Accordingly, functional enhancement and product safety should be considered simultaneously when developing different honey‐based foods and formulations. The combination of fruits (here: blackcurrants) and honey may therefore offer multiple advantages, including increased consumer appeal and the potential synergistic enhancement of antioxidant and antimicrobial properties. The latter aspect is particularly relevant for product stability. Despite this product potential, the technological approach used to enrich honey may critically determine the final functional and quality outcomes. Some studies primarily focused on the incorporation of dried or freeze‐dried fruits at relatively low supplementation levels, usually not exceeding 1%–4%. While these approaches demonstrated certain improvements in antioxidant parameters, they do not fully reflect the potential of less‐processed fruit matrices, such as frozen fruit purees, which may better preserve native phytochemicals and cellular microstructure, potentially affecting the release dynamics and interaction of bioactive compounds within the honey matrix. However, these studies were mainly about selected bioactive outcomes, while broader quality aspects remained underexplored.

The present study addressed these gaps by exemplarily applying frozen blackcurrant puree at higher incorporation levels, enabling a more comprehensive assessment of how minimally processed fruit ingredients influence honey quality, stability, antioxidant potential, and microbial characteristics. This approach extends current knowledge beyond studies relying on dehydrated fruit additives and provides new insights into the technological and functional implications of using frozen fruit matrices in honey enrichment. Therefore, this study aimed at investigating whether the addition of frozen fruit components at higher concentrations could improve the antioxidant properties of honey, while also assessing their impact on antimicrobial activity and product shelf‐life.

## 2 Materials and Methods

### 2.1 Research Material

As the basis, buckwheat honey ( *Fagopyrum esculentum* L.) was sourced from a local apiary in Poland's Podlasie region during the beekeeping season. Frozen blackcurrant ( *Ribes nigrum* ) berries of unknown cultivar were obtained from a local grocery store. The blackcurrants were thawed at 30°C for 90 min and then blended for 2 min, resulting in a smooth pulp. Three formulations were prepared by mixing ingredients in the following proportions: 97 g of honey and 3 g of blackcurrant pulp (HBC‐3), 93 g of honey and 7 g of blackcurrant pulp (HBC‐7), 88 g of honey and 12 g of blackcurrant pulp (HBC‐12). The pure honey served as a control sample. The selected supplementation levels were based on previous studies typically applying low fruit additions (1%–4%) in honey formulations (Grabek‐Lejko et al. 2022; Miłek et al. 2023) and were intentionally extended to higher concentrations enabling the assessment of dose‐dependent effects and potential technological limitations associated with increased fruit incorporation. The three levels were designed to represent low, intermediate, and high supplementation scenarios while maintaining practical processability of the honey matrix.

### 2.2 Physicochemical Analysis

#### 2.2.1 Free Acidity and pH

A solution was prepared by dissolving 10 g of honey in 75 mL of distilled water. pH value was recorded using a SevenCompact Duo S213 pH meter (Mettler‐Toledo GmbH, Greifensee, Switzerland). Free acidity was assessed as described by Bogdanov (2009), by titrating the solution with 0.1 M NaOH until a pH value of 8.3 was reached.

#### 2.2.2 Electrical Conductivity

Electrical conductivity of honey was determined using a solution of 20 g of dry matter dissolved in 100 mL of distilled water with a portable conductometer (CPC‐401, Elmetron Sp.j., Zabrze, Poland).

#### 2.2.3 Total Sugars

Total sugar content was measured using the lane‐eynon method, following the procedure described by Bogdanov (2009).

#### 2.2.4 Protein

Protein content was determined using the kjeldahl method. Samples of 1 g were digested with sulfuric acid and catalysts in a Büchi Wet Digester B‐426, along with a B‐414 scrubber (Büchi Labortechnik AG, Switzerland). After the digestion, a steam distillation was performed with automatic sodium hydroxide addition using the Büchi Distillation Unit K‐314 (Büchi Labortechnik AG, Switzerland). Tashiro's indicator was then added to the distillates, which were titrated with 0.1 M hydrochloric acid solution. The nitrogen content obtained was converted to protein using a conversion factor of 6.25.

#### 2.2.5 Total Phenolic Content (TPC)

Total phenolic content was assessed using a method initially outlined by Singleton et al. (1999), with minor modifications. A 1 g sample of honey was dissolved in 10 mL of distilled water. Next, 0.5 mL of this solution was combined with 2.5 mL of Folin–Ciocalteu reagent (0.2 N) and 2 mL of sodium carbonate solution (75 g/mL). The mixture was then incubated at 25°C in the dark for 2 h. Following the incubation, the absorbance was measured at 760 nm using a Nanocolor UV/VIS II spectrophotometer (Macherey‐Nagel GmbH & Co. KG, Düren, Germany). TPC was expressed as gallic acid equivalents (mg GAE/100 g).

#### 2.2.6 Radical Scavenging Activity

Radical scavenging activity was assessed following the method outlined by Turkmen et al. (2006), with slight modifications. Briefly, 2 g of the sample was dissolved in 10 mL of distilled water. Then, the solution was centrifuged at 10,000 × g and filtered. A 0.5 mL aliquot of the resulting solution was mixed with 1.5 mL of a 0.1 M DPPH solution in methanol. The sample was incubated at 25°C in the absence of light for 60 min. After incubation, the absorbance was measured at a wavelength of 517 nm using a Nanocolor UV/VIS II spectrophotometer (Macherey‐Nagel GmbH & Co. KG, Düren, Germany), with methanol serving as the blank. The control sample consisted of a mixture of the DPPH solution and distilled water. The radical scavenging activity (RSA%) was determined using the following equation:

$$\mathrm{RSA}\%=\left(\left(A_{0}-A_{a}\right)/A_{0}\right)\times 100,$$

where *A*_a_, the absorbance of the sample; *A*_0_, the absorbance of the control sample.

### 2.3 Microbiological Properties

For microbiological analysis, honey suspensions (5 g in 45 mL buffered peptone water 0.1%) were prepared (Rosiak et al. 2020; Sereia et al. 2017).

#### 2.3.1 Microbial Contamination

For the microbiological quality assessment, a reference agar plate method was selected. After homogenizing the prepared suspensions, samples (0.5 mL) were plated on microbiological culture media using the surface plating method. All microbiological tests were performed in triplicate. To detect and cultivate microorganisms present in honey's samples, the following microbiological media were used: standard plate count agar PCA (Argenta, Poland) – analysis of the total count of mesophilic aerobic bacteria, Malt Extract Agar (MEA) (BTL, Poland) – analysis of the yeast and molds, Baird‐Parker selective agar (Thermo Fisher Scientific, USA) – analysis of the presence of Gram‐positive bacteria *Staphylococcus aureus* , CN agar (Thermo Fisher Scientific, USA) for selective isolation of Gram‐negative bacteria *Pseudomonas aeruginosa*, Violet Red Bile Glucose (VRBG) medium (Thermo Fisher Scientific, USA) – analysis of fecal coliforms (*Enterobacteriaceae* family), and Tryptone Bile X‐Glucuronide (TBX) medium (BTL, Poland) for the selective detection and enumeration of *Escherichia coli*, fecal Gram‐negative bacteria.

After inoculation, Baird‐Parker, VRBG, and CN media were incubated at 37°C for 24–48 h, while PCA medium was incubated at 30°C for 48 h, MEA at 30°C for 7–10 days, only TBX agar needs incubation at 44°C for 24 h (ISO 2001). After incubation, the growth of bacteria and fungi on the media was observed. The number of microorganisms was counted using an automatic colony counter, model 7510/AES with the Easy Count 2 program (AES Chemunex SA, Marcy‐l'Etoile, France). Microbial counts were expressed as colony forming units per gram (CFU/g) of honey samples.

#### 2.3.2 Antibacterial Properties

Antibacterial activity was tested against selected bacterial strains using the agar well diffusion method. The antibacterial activity was evaluated against four reference bacterial strains: *Escherichia coli* ATCC 8739, *Staphylococcus aureus* ATCC 6538, *Bacillus subtilis* ATCC 6633, *Propionibacterium acnes* PM 2400. The bacterial inoculum of each strain was prepared in standardized 0.85% saline solution and adjusted to a concentration of 1.5 × 10^8^ CFU/mL (0.5 McF on the McFarland scale) based on densitometric measurements using Densimat (bioMérieux). The bacterial suspensions were inoculated onto Muller‐Hinton agar (BTL, Poland). The honey samples were introduced into the wells, which were cut in the media and incubated at 37°C for 18–24 h. The antibacterial activity was indicated by the presence of a growth inhibition zone around the well after incubation (Delavault et al. 2022).

#### 2.3.3 Microbial Stability During Short‐Term Storage—Challenge Test

To determine whether the addition of blackcurrant puree reduced honey's natural resistance to microbial growth, the honey samples (without and with different content of blackcurrant puree) placed in sterile containers were inoculated with suspensions of selected microorganisms. The survival of aerobic spore‐forming Gram‐positive bacterium *Bacillus subtilis* and osmotolerant, xerotolerant and halophilic molds *Penicillium chrysogenum*, which are commonly found in food products and indoor environments, were tested.

The *Bacillus subtilis* bacterial growth was suspended in standardized 0.85% saline solution and adjusted to a concentration of 1 × 10^7^–1 × 10^8^ CFU/mL based on densitometric measurements using Densimat (bioMérieux). The *Penicillium chrysogenum* fungal suspension was prepared from a 7‐day culture grown on potato dextrose agar (PDA) (BTL, Poland) at 30°C. The fungal spores were suspended in standardized 0.85% saline solution shaken for 10 min at 200 rpm to homogenize, and adjusted to a concentration of 1 × 10^6^–1 × 10^7^ CFU/mL based on microscopic counts using a Bürker chamber (BLAUBRAND, Germany). The initial amount of bacteria or fungal spores in the prepared inoculum was verified using agar plate methods.

The prepared microorganism suspensions were put in samples of honeys (5 g), mixed, and stored at room temperature for 2 weeks. To assess differences in the survival of bacteria and fungi in the samples depending on fruit content, each sample was tested immediately after the microorganisms inoculation and after the 14‐day storage, using the agar plate method. For each sample at both time points, initial suspensions (2.3.1) and decimal serial dilutions were prepared. Plate count agar—PCA (Argenta, Poland) and potato dextrose agar—PDA (BTL, Poland) were used to enumerate microorganisms. After incubation *Penicillium chrysogenum* (30°C/7 day) and *Bacillus subtilis* (37°C/48 h), the number of colonies grown on the media were counted using an automatic colony counter model 7510/AES with the Easy Count 2 program (AES Chemunex SA, Marcy‐l'Etoile, France), with statistical correction and calculated as colony‐forming units (CFU) per gram of honey. Differences in the survival ability of bacteria and fungi in various honey formulations with a fruit component were expressed on a logarithmic scale as the degree of microorganism count reduction.

### 2.4 Sensory Analysis

To assess the desirability of the prepared buckwheat honey samples with varying amounts of blackcurrant puree, a sensory analysis was conducted, evaluating color, consistency, aroma, taste, and overall desirability. A group of 64 consumers, aged between 19 and 80 years, participated in the tests, including 32 women (23–80 years) and 32 men (19–74 years). All participants were regular consumers being familiar with honey products. The sensory evaluation was carried out according to ISO (2003) guidelines. The variables being assessed were explained to the participants during a training session. A seven‐point hedonic scale was used for evaluation, with 7 representing “extremely like” and 1 representing “extremely dislike”. All sensory evaluations were performed under artificial daylight in a sensory lab designed to meet ISO (2007) standards. The room temperature was maintained between 20°C and 22°C with air circulation. To prevent flavor contamination between samples, assessors were provided with 50 mL of plain water. All participants provided written informed consent prior to participation. The study was conducted in accordance with the Declaration of Helsinki and complied with the ethical standards adopted by the Krakow University of Economics (Senate Resolution No. 38/2011).

### 2.5 Statistical Analysis

The data were analyzed using statistical methods with R 4.4.1 software (R Core Team 2024), with a significance level of 0.05. Prior to statistical analysis, data distribution was assessed using the Shapiro–Wilk test to verify normality assumptions. As the assumptions of normal distribution were not met, nonparametric methods were applied. The Kruskal‐Wallis test was used to compare quantitative variables across the four groups. When significant differences were identified, Dunn's post hoc test was conducted to pinpoint the specific samples that differed. Principal component analysis (PCA) was also performed to create a 2‐dimensional map, reducing dimensionality and highlighting differences between the honey samples containing various amounts of blackcurrant puree and the control sample.

## 3 Results and Discussion

### 3.1 Physicochemical and Antioxidant Properties

Although Polish buckwheat honey is known for its high bioactivity, blackcurrant puree was added to further enhance its properties, aligning with key factors driving honey selection, such as health benefits and sensory qualities (Sparacino et al. 2022). The obtained values were evaluated with regard to compositional criteria defined for honey in EU legislation (Council Directive 2001/110/EC 2002), serving as a reference framework for assessing changes within the honey matrix.

The addition of blackcurrant resulted in a pronounced acidification of the formulations (Table 1), reflected by a higher free acidity and a corresponding decrease in pH, most likely due to organic acids introduced with the fruit ingredient. While the control sample and the formulation HBC‐3 (honey with 3% blackcurrant puree) remained within the acidity limits specified for honey, formulations with higher blackcurrant content exceeded these thresholds, indicating that such products cannot be considered as legally‐defined honey anymore and need to be labeled as honey‐based products or formulations.

**TABLE 1:** Main physicochemical parameters of the HBC products, depending on the amount of added blackcurrant. Results given as mean ± standard deviation, *n* = 3.

Parameter	Sample				*p*
	C	HBC‐3	HBC‐7	HBC‐12	
pH	3.93 ± 0.19^a^	3.76 ± 0.07^a^	3.55 ± 0.05^ab^	3.47 ± 0.04^b^	0.022*
Free acidity (mEq/kg)	33.33 ± 1.53^c^	45.67 ± 0.58^b^	64.67 ± 1.53^ab^	88.33 ± 0.58^a^	0.015*
Conductivity (μS/cm)	210 ± 1^c^	217 ± 4^b^	239 ± 2^ab^	278 ± 3^a^	0.014*
Protein (g/100 g)	0.45 ± 0.05^b^	0.49 ± 0.04^b^	0.52 ± 0.01^ab^	0.55 ± 0.01^a^	0.022*
Total sugar content (g/100 g)	80.77 ± 0.36^a^	75.58 ± 0.16^ab^	71.83 ± 0.14^bc^	67.44 ± 0.12^c^	0.015*
TPC (mg GAE/100 g)	126.52 ± 4.42^b^	133.1 ± 6.03^b^	140.56 ± 6.97^ab^	152.86 ± 6.21^a^	0.027*
RSA (%)	56.9 ± 2.43^b^	77.11 ± 0.56^ab^	81.42 ± 0.45^a^	82.52 ± 1.07^a^	0.021*

*Note:* Different superscript letters within a row indicate statistically significant differences between samples.

* Statistically significant difference (*p* < 0.05).

The supplementation also increased conductivity. A significant increase was observed in samples HBC‐7 and HBC‐12. However, all the samples still met the requirements for the definition of blossom honey.

Among different types of honey, the control honey exhibited a notably high protein content (da Azeredo et al. 2003). However, supplementation led to a slight increase in protein concentration, which was statistically significant only in HBC‐12.

Moreover, the supplementation caused a decrease in total sugar content (from 80.8 g/100 g in the control sample to 67.4 g/100 g in the HBC 12 sample). In samples with 7% and 12% supplementation, this drop was statistically significant. Such a lowering may be beneficial in certain circumstances, potentially enhancing the product's suitability for individuals aiming to reduce their sugar intake (Tomczyk et al. 2020). Furthermore, it should be noted that a specific sugar‐to‐acid ratio may be preferred by consumers (Halagarda and Suwała 2018). Therefore, the observed decrease in sugar content and a parallel increased acidity may be beneficial or unfavorable depending on the balance between these parameters, emphasizing the need for further research to identify the most desirable composition.

As intended, the use of blackcurrant puree enhanced the antioxidant properties of the honey. Total phenolic content increased from 126.5 mg GAE/100 g (in the control sample) to 152.9 mg GAE/100 g (in the HBC 12 sample), though the increase was statistically significant only in the sample with 12% blackcurrant puree. At the same time, radical scavenging activity increased from 56.9% in the control sample to 82.5% in the HBC 12 sample. A statistically significant increase was observed in samples containing 7% and 12% blackcurrant puree. The enhancement of honey's antioxidant properties following fruit addition is attributed to synergistic interactions between honey's antioxidants and those found in plant material, primarily polyphenols and vitamin C (Grabek‐Lejko et al. 2022). Similarly, Miłek et al. (2023) reported enhanced antioxidant activity in rapeseed honey after supplementation with freeze‐dried blackcurrant (2% and 4%), as did Grabek‐Lejko et al. (2022) following the addition of dried and freeze‐dried blackcurrant (1% and 4%). However, the effect observed in their studies was more pronounced, likely due to the use of dehydrated fruits or a different food matrix (rapeseed honey).

### 3.2 Principal Component Analysis (PCA) Concerning Physicochemical and Antioxidant Properties

To provide an integrated view of the relationships among the measured physicochemical parameters and to visualize similarities and differences between samples, principal component analysis (PCA) was performed. PCA was used as an exploratory multivariate tool to reduce data dimensionality and identify the main variables responsible for sample differentiation.

**FIGURE 1:**
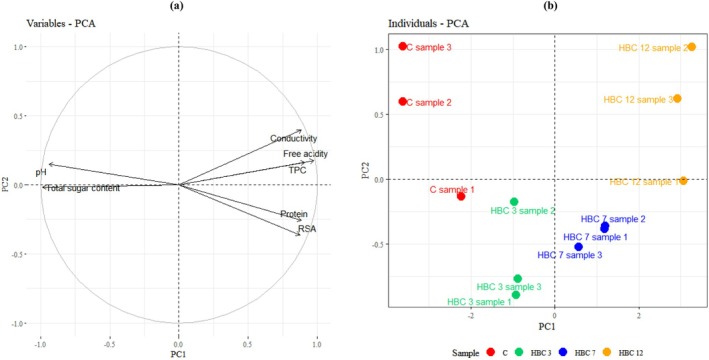
Comparison of the analyzed samples based on principal component analysis (PCA). (a) Variables factor map; (b) Individuals factor map.

The first two PC accounted together for 91.44% of the total variance. The first component explained 85.18%, while the second contributed 6.26% (Figure 1a).

The individuals factor map shows a clear grouping of samples along the first component (Figure 1b), reflecting the effect of increasing blackcurrant puree addition. Samples with higher puree content were associated with a lower pH value and lower total sugar content, as well as higher values of the remaining parameters, confirming the overall compositional shift observed in the individual parameter analyses.

### 3.3 Microbiological Properties

#### 3.3.1 Microbial Contamination

The microbiological contamination of honey after being supplemented with a fruit component was assessed. The tests were focused mainly on detecting culturable heterotrophic mesophilic (aerobic and facultative anaerobic) bacteria, yeast, and molds, as well as the presence of fecal coliforms and some potentially pathogenic bacteria, which determined the microbial quality of food products (Fernandez et al. 2017). The results of the analysis are presented in Table 2.

**TABLE 2:** The microbiological contamination of honey with an addition of blackcurrant puree. Results given as mean.

Type of microbiological indicator	Sample			
	C	HBC‐3	HBC‐7	HBC‐12
Total count of microorganisms (TCMA) (CFU/g)	5.0 × 10^1^	8.0 × 10^1^	1.2 × 10^2^	5.0 × 10^1^
*Staphylococcus aureus* (CFU/g)	Not detected	Not detected	Not detected	Not detected
*Pseudomonas aeruginosa* (CFU/g)	Not detected	Not detected	Not detected	Not detected
Fecal coliforms (*Enterobacteriaceae*) (CFU/g)	Not detected	Not detected	Not detected	Not detected
*Escherichia coli* (CFU/g)	Not detected	Not detected	Not detected	Not detected
Yeasts and molds (CFU/g)	2.0 × 10^1^	Not detected	1.0 × 10^1^	Not detected

From the prepared samples of buckwheat honey and honey with added blackcurrant puree, heterotrophic mesophilic bacteria, as well as yeast and molds, were isolated. The total count of microorganisms per 1 g of honey ranged from 5.0 × 10^1^–1.2 × 10^2^ CFU, depending on the sample, which can be considered a comparable result to other studies (Luca et al. 2025; Grabek‐Lejko and Worek 2024; Kędzierska‐Matysek et al. 2023; Fernandez et al. 2017). In 163 honey samples obtained from different processing points in the Pampas Region (Argentina), the total number of bacteria did not exceed 5.5 × 10^1^ CFU/g (Fernandez et al. 2017). In some research, Grabek‐Lejko and Worek (2024) in honeydew honey collected in Poland, the total number of bacteria did not exceed the value of 2.5 × 10^2^ CFU/mL, and *Bacillus pumilus/alitudinis, B. licheniformis*, and *Bacillus cereus* groups were the dominant identified bacteria. Kędzierska‐Matysek et al. (2023) evaluated the quality and food safety of Polish varietal honeys. The study included 21 honey samples from five varieties (multifloral, honeydew, rapeseed, buckwheat, and linden), and more than half of the analyzed honeys contained fewer microorganisms than 10 CFU/g, but in the rest of the samples, the colony count of the presumptive *Bacillus* spp. ranged from 5.0 × 10^1^—4.5 × 10^2^ CFU/g. Moreover, in these studies, the anaerobic spore‐forming bacteria and fungi were detected only in a sample of buckwheat honey (Kędzierska‐Matysek et al. 2023). Microscopic analysis of the cell morphology from the grown colonies in our study similarly confirmed the presence of spore‐forming and nonspore‐forming bacilli, as well as yeast. It is related to the physicochemical parameters of honey (like high sugar content, low pH, and low water content) and biological origin, which make it an ideal substrate for the development of xerotolerant fungi and promote surviving spore‐forming bacteria and osmophilic yeasts (Rosiak et al. 2020; Rodríguez‐Andrade et al. 2019). Among the yeasts, the most prevalent species are *Hansenula*, *Nemaiospora*, *Pichia*, *Rhodotorula*, *Saccharomyces*, *Schizosaccharomyces*, *Torula*, and *Torulopsis*. Yeasts are often determined together with molds because they show resistance to a high osmotic pressure caused by low water activity (Rosiak et al. 2020; Snowdon and Cliver 1996). Most of the obligate xerophiles and xerotolerant fungal strains, which have been reported in honey, belong to the ascomycetous genera *Aspergillus*, *Bettsia*, *Candida*, *Eremascus*, *Monascus*, *Oidiodendron*, *Penicillium*, *Skoua*, *Talaromyces*, and *Zygosaccharomyces* (Rodríguez‐Andrade et al. 2019). In the present study, only single colonies of fungi were detected in control honey and HBC‐7 sample. Among the microorganisms detected on the culture media, only *Penicillium* sp. was identified.

The microbial contamination tests did not confirm the presence of pathogenic bacteria such as *Staphylococcus aureus* and *Pseudomonas aeruginosa* , for which selective media were used. Additionally, no bacteria from the *Enterobacteriaceae* family, including *Escherichia coli* , were determined. These are typical hygienic indicators during food production. The microbiological tests did not show any sanitary risks.

**FIGURE 2:**
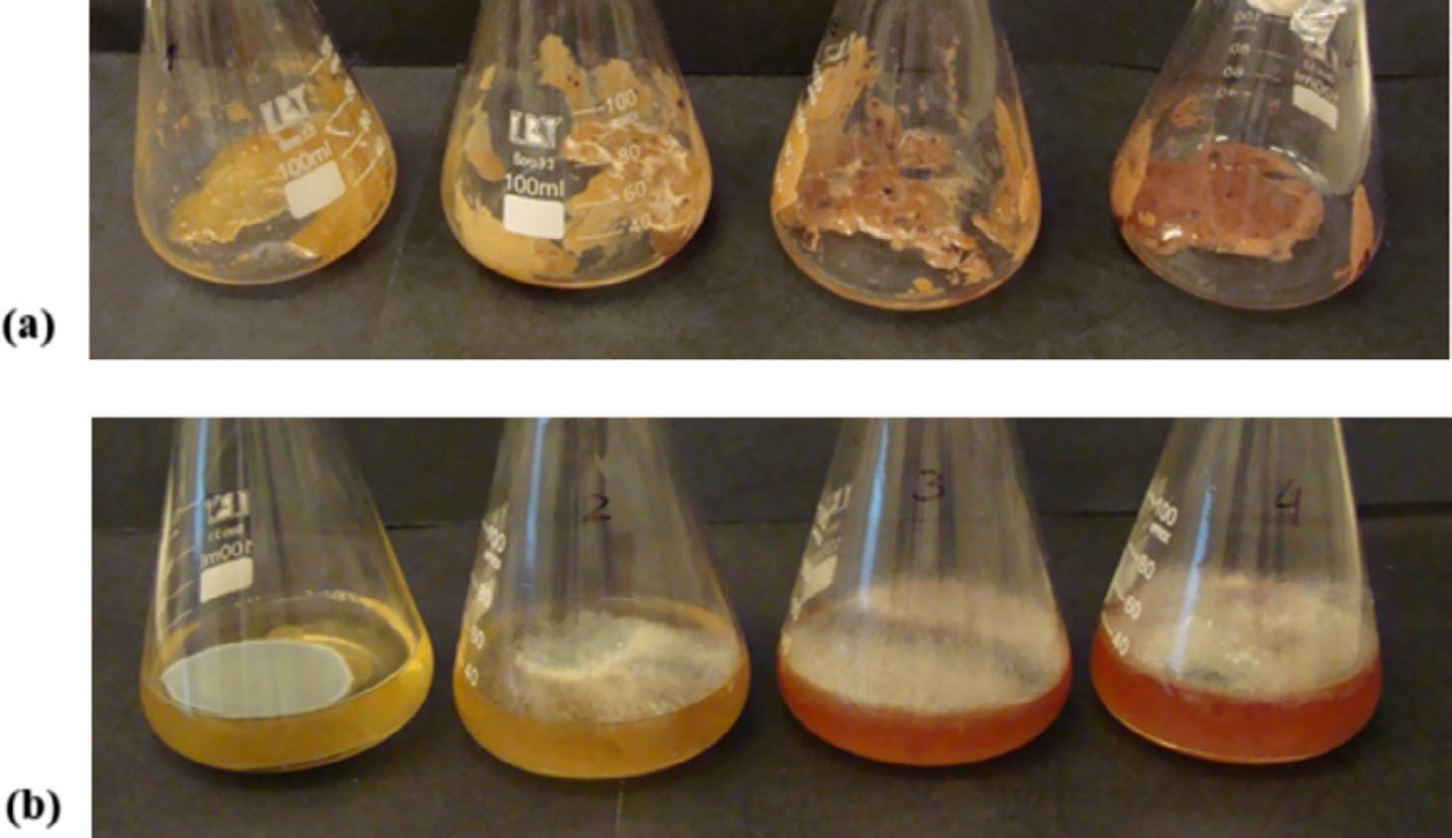
Weighed samples (a) and initial suspensions of honey samples with fruit additives in peptone saline after 10 days from preparation (b) (from left to right: Samples C, HBC‐3, HBC‐7, HBC‐12).

Additionally, before conducting the cultivation‐based tests, the honey samples prepared with the addition of blackcurrant puree were observed in terms of the development of microbiological contaminations during storage. After weighing, they were stored under stable conditions at a temperature of 22°C ± 2°C in sterile containers for 2 weeks. During this storage period, no growth of microorganisms or other signs of spoilage were observed on the samples (Figure 2a), indicating that neither the honey itself nor the addition of blackcurrant puree negatively affected the microbiological stability of the product.

The lack of visible microbial growth during the preliminary storage period confirmed an appropriate microbiological stability of the honey‐fruit formulation. This further suggested that the inherent physicochemical properties of the initial pure honey, combined with the quality of the blackcurrant puree, effectively inhibit the proliferation of spoilage organisms under standard storage conditions. However, the addition of buffered peptone water to prepared initial suspensions from honey, in the next step of the experiment, led to an increase in water activity (*a*_w_) and a reduction in high osmotic potential (a property that typically protects honey from microbial growth). It allowed for the detection of fungal contamination (Figure 2b). It is important to note that fungal spore contamination from the environment can occur at any stage of honey production and storage, as it is a naturally derived product. Furthermore, the addition of buffered peptone water in the microbiological test may facilitate the activation of xerophilic fungi that are inherently present in honey as well as blackcurrant puree. These observations suggested that while the raw honey mixture appears stable, the transition to a high water activity environment caused by both the fruit addition and the analytical dilution process may trigger the activation of xerophilic fungi, while the overall raw honey mixture appeared to be stable.

**FIGURE 3:**
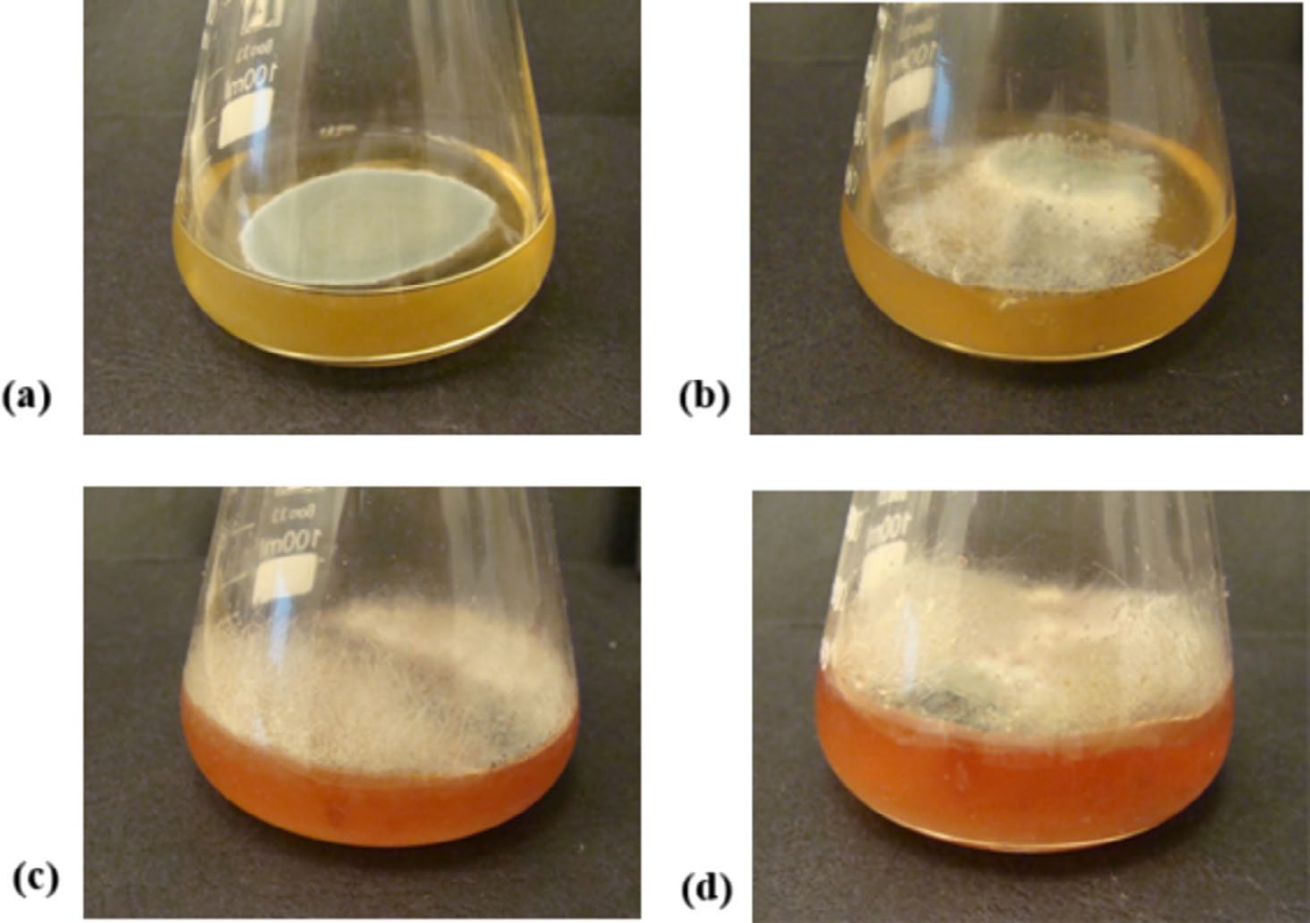
Development of *Penicillium* sp. in honey suspensions with fruit components in samples (a) C and (b) HBC‐3, and fungi from the Zygomycota phylum in samples (b) HBC‐3, (c) HBC‐7, and (d) HBC‐12.

The two‐week observation of initial suspensions prepared from individual samples and stored at a temperature of 22°C ± 2°C revealed the presence of mold fungi (Figure 3). In samples HBC‐3, HBC‐7, and HBC‐12, fungi recognized as representants of *Mucorales* were detected as early as the third day of observation. These fast‐growing fungi are a common cause of spoilage in vegetables and fruits, including those stored frozen (Rodríguez‐Andrade et al. 2019; Kwinda et al. 2015). The control honey sample initially showed no signs of contamination, but after 7 days, an active growth of *Penicillium* sp. occurred. Fungi from the genus *Penicillium* are ubiquitous molds found in the natural environment and production areas. Therefore, it is difficult to determine the source (primary or secondary) and timing of contamination with them. The presence of *Mucorales* only in honey samples with blackcurrant additives seems to suggest that the contamination may have originated from the fruit component. Both filamentous fungi—*Penicillium* and *Mucor*—are commonly found in plant pollen, transmitted to honeybees and frequently found in bee bread (Disayathanoowat et al. 2020; Xiong et al. 2023), which in turn suggests the primary source of contamination. However, it is important to note that the development of these fungi did not occur in the watered‐down samples before preparing the suspension/mixing with buffered peptone water, which are naturally available to consumers. However, these results demonstrated that primary and secondary sources of contamination may be responsible for the decrease in honey quality.

Although no strictly pathogenic microorganisms were detected, the rapid growth of *Mucorales* and *Penicillium* spp. following sample hydration raised significant concerns regarding the product's long‐term stability and consumer safety. The presence of these fungi suggested a higher potential for secondary spoilage. Although the low water activity of raw honey currently acts as a preservative, any incidental increase in moisture during consumer use could trigger the germination of these spores, leading to rapid quality degradation (sensory quality) and potential exposure to toxic (mycotoxins) and nontoxic metabolites.

#### 3.3.2 Antibacterial Properties

**FIGURE 4:**
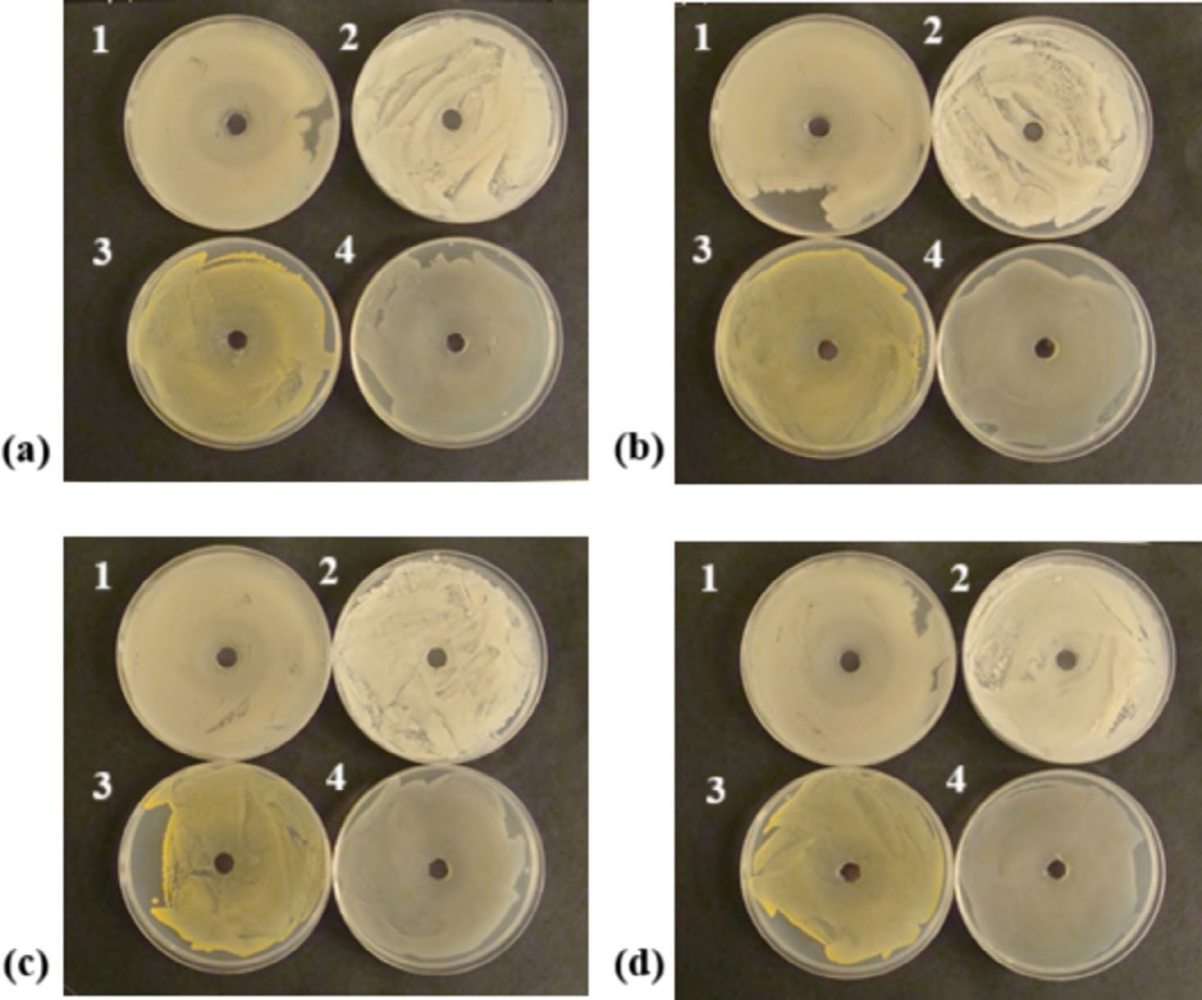
Assessment of antibacterial activity of honey with fruit components using the agar diffusion method; (a) Sample C, (b) Sample HBC‐3, (c) Sample HBC‐7, (d) Sample HBC‐12; 1— *Propionibacterium acnes* , 2— *Bacillus subtilis* , 3— *Staphylococcus aureus* , 4— *Escherichia coli* .

Observations of bacterial cultures conducted in the presence of honey with fruit components did not reveal any antibacterial activity of the honey formulations tested with different agar diffusion methods. No inhibition zones were observed around the wells into which honey suspensions in peptone saline were given for the test bacteria, namely *Staphylococcus aureus* , *Escherichia coli* , *Propionibacterium acnes* , and *Bacillus subtilis* , indicating a lack of antibacterial activity of the honey against these prominent organisms (Figure 4). However, this effect may have been influenced by the dilution and hydration of the product samples. The agar diffusion method was unable to demonstrate any antibacterial activity in the tested samples or to compare whether the addition of fruit component had an impact on diminishing this effect. Thus, the lack of a visible inhibition zone in the present study may originate from the physicochemical constraints of the method rather than a lack of antimicrobial potential in the blackcurrant honey. The high viscosity and osmotic pressure of the samples likely impeded the effective migration of bioactive compounds into the agar, suggesting that alternative methods, such as broth microdilution, might be more suitable for evaluating the potency of such dense matrices (Luca et al. 2025).

With regard to blackcurrants, it was expected that the antibacterial activity of honey might increase. Blackcurants have been described as having potent antiviral and anti‐bacterial activities (Ikuta et al. 2012). In this context, Miłek et al. (2023) already observed that rapeseed honey exhibited antibacterial activity against *S. aureus* and *Klebsiella pneumoniae* after the addition of freeze‐dried blackcurrant (4%). The authors attributed this effect to the enrichment of honey with the presence of berry anthocyanins. However, although anthocyanins are well‐known to provide a certain activity, even antibacterial properties in model approaches (Ma et al. 2019), it seems to be more feasible that phenolic compounds that are more reactive at all, might also provide more antibacterial activity. Here, especially phenolic acids can be mentioned. Caffeic acid is one of the most reactive phenolic compounds at all. Also with regard to antibacterial activity, it is quite bioactive (Kępa et al. 2018).

#### 3.3.3 Preservation Test

**FIGURE 5:**
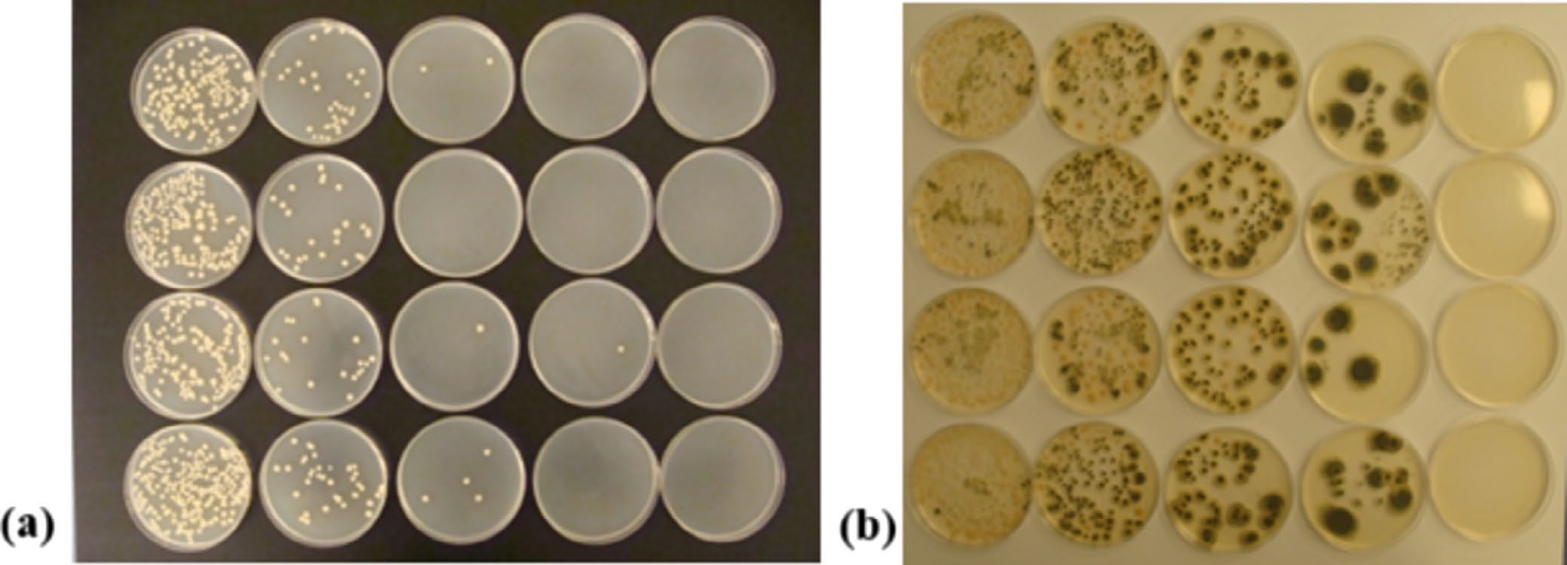
Growth of *Bacillus subtilis* (a) and *Penicillium chrysogenum* (b) in samples' cultures after a 2‐week storage period following inoculation (from top to bottom – samples: C, HBC‐3, HBC‐7, HBC‐12), plates from left to right show dilutions from 100 to 10^−4^ mL of extracts.

It was further studied whether suspensions of selected microorganisms, *Bacillus subtilis* and *Penicillium chrysogenum*, which were intentionally introduced into honey samples, could survive in honey during storage and testing if an addition of fruit might increase the survival of these microorganisms, while simultaneously reducing the honey's microbiological stability. The changes in numbers of bacteria and fungi during storage were monitored. The secondary contamination test of samples (Table 3) did not show significant changes in the number of bacteria during storage for any of the formulations studied. The calculated reduction logarithms did not indicate any substantial differences in the number of *Bacillus subtilis* throughout the challenge test for the individual formulations or between the honey samples (Figure 5). Thus, the addition of the fruit component did not increase or decrease the honey's preservative properties against bacterial growth. However, honey's initial protective properties against fungal growth were not as strong. During storage, a more than tenfold to nearly hundredfold increase in the number of fungi was observed. For these microorganisms, no impact of the fruit component on reducing microbiological stability was noted in comparison to the samples without the blackcurrant addition. Molds tolerate osmotic stress better than most bacteria (Pitt and Hocking 2009).

**TABLE 3:** The impact of blackcurrant puree addition on the reduction of honey's preservation ability against microbiological spoilage. Results given as mean.

Sample	*Bacillus subtilis* bacteria in samples just after inoculation (*t*_0_) (CFU/g)	*Bacillus subtilis* bacteria in samples 2 weeks after inoculation (*t*_2weeks_) (CFU/g)	*Penicillium chrysogenum* fungi in samples just after inoculation (*t*_0_) (CFU/g)	*Penicillium chrysogenum* fungi in samples 2 weeks after inoculation (*t*_2weeks_) (CFU/g)
C	1.34 × 10^6^	1.04 × 10^6^	2.76 × 10^4^	9.15 × 10^5^
	log = 6.13	log = 6.02	log = 4.44	log = 5.96
	*R* = 0.11		*R* = (−1.52)	
HBC‐3	0.88 × 10^6^	0.15 × 10^6^	5.27 × 10^4^	9.25 × 10^5^
	log = 5.94	log = 5.18	log = 4.72	log = 5.97
	*R* = 0.76		*R* = (−1.25)	
HBC‐7	0.84 × 10^6^	0.55 × 10^6^	2.52 × 10^4^	4.75 × 10^5^
	log = 5.92	log = 5.74	log = 4.40	log = 5.68
	*R* = 0.18		*R* = (−1.28)	
HBC‐12	1.13 × 10^6^	2.12 × 10^6^	1.51 × 10^4^	11.05 × 10^5^
	log = 6.05	log = 6.32	log = 4.18	log = 6.04
	*R* = (−0.27)		*R* = (−1.86)	

The relevance of these challenge tests partially extends to real consumer handling, where honey is frequently exposed to fluctuating environmental conditions and secondary contaminations. The test results confirm that while the honey samples remain resistant against bacterial proliferation ( *Bacillus subtilis* ), its natural protection against molds is more fragile. In a consumer context, even a slight localized increase in moisture could lower the osmotic pressure enough to allow introduced fungal spores, such as Penicillium chrysogenum, to germinate and elevate the microbial risk. The observed 10‐to‐100‐fold increase in fungal counts during the challenge test highlights that maintaining low water activity through proper sealing and dry handling of the product is still the critical point.

### 3.4 Sensory Analysis

Sensory characteristics of a food product significantly influence its consumer acceptance (Kardas et al. 2024; Laaksonen et al. 2016), often taking precedence over potential health benefits (Laaksonen et al. 2016). Sensory properties of honey are closely linked to its botanical origin (Kardas et al. 2024; Kortesniemi et al. 2018). Dark‐colored honeys, such as buckwheat honey, exhibit the strongest antioxidant potential (Wesołowska and Dżugan 2017; Dżugan et al. 2018; Pentoś et al. 2020; Starowicz et al. 2021). However, due to their color and distinctive stronger and more robust flavor (Šedík et al. 2023), they are the least preferred by consumers (Kortesniemi et al. 2018). Similarly, blackcurrant ( *Ribes nigrum* ) berries, due to their sour taste and astringent mouthfeel, are perceived by many consumers as unpleasant (Väkeväinen et al. 2021; Laaksonen et al. 2016). However, Šedík et al. (2023) reported that honey can become more appealing when combined with various additions, whereas according to Väkeväinen et al. (2021) blackcurrants are better perceived when sweetened.

The results of the consumer desirability test in the present study confirm that selected sensory parameters of the buckwheat honey improve with the addition of blackcurrant puree, enhancing its attractiveness to consumers (Table 4). Considering the observed physicochemical changes, particularly the reduction in sugar content and the increase in acidity following blackcurrant addition, the incorporation of blackcurrant puree likely influenced the perceived balance between sweetness and sourness, which is known to play a crucial role in consumer acceptance (Laaksonen et al. 2016). The low supplementation level (HBC‐3) may have produced an insufficient sensory shift, resulting in a product that combined the strong flavor profile of buckwheat honey with only a slight acidic modification, which could explain its lower desirability. In contrast, higher supplementation (HBC‐12), associated with a more pronounced decrease in sugars and increased acidity, may have generated a more harmonious sensory balance, reducing the characteristic intensity of buckwheat honey and enhancing overall consumer perception.

**TABLE 4:** Results of sensory analysis, depending on the addition of blackcurrant, evaluated using a seven‐point hedonic scale. Results given as mean ± standard deviation.

Parameter	Sample	Hedonic score	*p*
Color	C	4.19 ± 1.20^b^	< 0.001*
	HBC‐3	2.84 ± 1.05^c^	
	HBC‐7	4.19 ± 1.28^b^	
	HBC‐12	5.25 ± 1.44^a^	
Texture	C	4.38 ± 1.1	0.493
	HBC‐3	4.25 ± 1.22	
	HBC‐7	4.34 ± 1.12	
	HBC‐12	4.62 ± 1.18	
Odor	C	4.28 ± 0.99^a^	0.003*
	HBC‐3	3.5 ± 0.98^b^	
	HBC‐7	4.09 ± 1.3^a^	
	HBC‐12	4.59 ± 1.27^a^	
Palatability	C	4.88 ± 1.13	0.255
	HBC‐3	4.53 ± 1.22	
	HBC‐7	4.59 ± 1.24	
	HBC‐12	4.97 ± 1.53	
Overall desirability	C	4.78 ± 1.01^a^	0.005*
	HBC‐3	4.22 ± 0.94^b^	
	HBC‐7	4.22 ± 1.04^b^	
	HBC‐12	4.97 ± 1.31^a^	

*Note:* Different superscript letters indicate statistically significant differences among samples within the same sensory parameter (*p* < 0.05).

* Statistically significant difference (*p* < 0.05).

Moreover, it was shown that honey with a 12% blackcurrant puree (HBC‐12) and pure honey (C) were judged to be the most desirable overall. The smell of these samples was also rated the highest, while HBC‐12 had the best‐rated color. Conversely, the sample with a 3% blackcurrant puree (HBC‐3) received the lowest ratings for color, odor, and overall desirability. Consumers generally disliked its color and had a neutral opinion of its odor. Notably, all tested samples were equally appreciated for their texture and palatability, which according to Kortesniemi et al. (2018), influence consumer preference for honey. In contrast, Ćetković et al. (2014) observed improved density and flavor but no significant effect on the aroma and color of linden honey after the addition of dried apricots. However, all modified honey samples were assessed as being of very good sensory quality.

## 4 Conclusions

The outcomes of this study demonstrate that incorporating blackcurrant puree into Polish buckwheat honey significantly enhances its antioxidant properties, while altering its physicochemical characteristics. The increased TPC and radical scavenging activity, particularly in the sample with 12% blackcurrant addition, highlight the potential of fruit supplementation to boost the functional value of honey. Furthermore, the observed decrease in sugar content, alongside increased acidity, may make the product more appealing to health‐conscious consumers. However, achieving an optimal sugar‐to‐acid ratio remains crucial, as this balance directly influences both sensory quality and consumer desirability.

Sensory evaluation confirmed that supplementation positively influenced product appeal, with the highest desirability observed for the sample containing 12% blackcurrant. This finding aligns with previous reports indicating that fruit addition can mitigate the intense sensory attributes of dark honeys, making them more attractive to consumers. Nevertheless, the study also revealed potential challenges regarding microbiological stability. Although the product exhibited acceptable microbiological purity under standard conditions, hydration during preparation facilitated the detection of environmental microorganisms, suggesting that the fruit component may introduce a contamination risk when not properly controlled.

From a regulatory perspective, the incorporation of blackcurrant puree alters the legal classification of the obtained formulations under current EU legislation for honey (Council Directive 2001/110/EC 2002), as products containing added ingredients are not allowed to be marketed as honey anymore, but need to be labeled as “honey‐based” or “honey‐like” products. Using compositional criteria defined for honey as a reference framework allowed the extent of physicochemical modifications within the honey matrix to be evaluated, showing that only the formulation containing 3% blackcurrant puree remained within the acidity limits established for honey, whereas higher supplementation levels exceeded these thresholds, confirming a dose‐dependent impact of fruit addition on product composition.

Further research on long‐term storage stability and consumer acceptance under real‐market conditions would provide valuable insights into the product's commercial potential. While blackcurrant‐supplemented honey‐based products/formulations offer promising health benefits and sensory appeal, their successful commercialization will require consideration of production challenges related to microbiological stability.

## Author Contributions

**Katarzyna Kowa‐Halagarda:** investigation, writing – original draft, formal analysis. **Marcin Laskoś:** investigation, formal analysis. **Michał Halagarda:** conceptualization, funding acquisition, methodology, writing – original draft, formal analysis, investigation, project administration, supervision. **Justyna Syguła‐Cholewińska:** methodology, investigation, writing – original draft. **Sascha Rohn:** conceptualization, funding acquisition, writing – review and editing, supervision.

## Funding

This research was subsidized by the Krakow University of Economics, Poland, project numbers 50/ZJZ/2024/DOS, 018/ZJB/2025/DOS.

## Conflicts of Interest

The authors declare no conflicts of interest.

## Data Availability

The data that support the findings of this study are available from the corresponding author upon reasonable request.
